# Three-Dimensional Morphometric Analysis of the Lisfranc Joint and Its Relationship to Injury

**DOI:** 10.3390/diagnostics16091264

**Published:** 2026-04-23

**Authors:** Cemre Savaşan, Abdul Veli İsmailoğlu, Samir İlgaroğlu, Edip Yılmaz, Alp Bayramoğlu

**Affiliations:** 1 Department of Anatomy, School of Medicine, Acibadem Mehmet Ali Aydinlar University, Istanbul 34752, Turkey; alp.bayramoglu@acibadem.edu.tr; 2 Department of Anatomy, Faculty of Medicine, Marmara University, Istanbul 34854, Turkey; abdulveli.ismailoglu@gmail.com; 3 Department of Orthopaedics and Traumatology, Faculty of Medicine, Marmara University, Istanbul 34854, Turkey; doktorsamir.zeynal@gmail.com; 4 Department of Orthopaedics and Traumatology, Acibadem Altunizade Hospital, Istanbul 34662, Turkey; edip19_87@hotmail.com

**Keywords:** Lisfranc joint injury, bone morphology, computed tomography, three-dimensional reconstruction, risk factor

## Abstract

**Background/Objectives:** Lisfranc joint injuries are complex midfoot pathologies frequently associated with subtle radiologic findings and delayed diagnosis. Although ligamentous disruption is considered the primary mechanism, the contribution of intrinsic osseous morphology remains insufficiently investigated. Previous studies have primarily relied on two-dimensional measurements and limited morphometric parameters. Therefore, this study aimed to provide a comprehensive three-dimensional (3D) computed tomography (CT) based morphometric evaluation of the medial and central columns of the Lisfranc joint and to determine whether specific bony parameters are associated with injury predisposition. **Methods:** A total of 48 CT scans, including 23 from patients with Lisfranc joint injuries and 25 from healthy controls without midfoot trauma, were retrospectively analyzed. For both groups, 3D models of the first three metatarsals (M1–M3) and cuneiforms (C1–C3) were reconstructed to measure bone length, articular surface areas, volumes, M1–M2/M2–M3 depth differences, and dorsal step-off (dorsal subluxation of M2 relative to C2). Correlations of these measurements with M2 length were additionally assessed in each group. **Results:** Comparisons between injury and healthy control groups revealed no significant differences in bony morphometrics (*p* > 0.05). Correlation analysis showed that a longer M2 were associated with greater cuneiform volumes and larger metatarsal articular surface areas (*p* < 0.05). **Conclusions:** This comprehensive 3D morphometric assessment of the Lisfranc joint indicates that intrinsic bony anatomy alone is unlikely to represent a primary predisposing factor for Lisfranc injuries. The observed positive relationship between M2 length and cuneiform articular surface areas and volumes demonstrates structural interdependence within the medial and central columns. Overall, injury susceptibility does not appear to be explained by variations in osseous morphology alone.

## 1. Introduction

The tarsometatarsal (TMT) joint, also known as the Lisfranc joint, is a complex joint between the tarsal and metatarsal bones that provides stabilization to the midfoot [1,2]. The term “Lisfranc” originates from Jacques Lisfranc de Saint-Martin (1787–1847), a French surgeon and gynecologist who served in Napoleon Bonaparte’s army and was the first to describe amputation through this joint after gangrene in a cavalry officer. Since then, the eponym has been used to refer to the joint between the tarsal and metatarsal bones [3,4,5,6]. From an anatomical and functional perspective, the joint is commonly described in terms of three columns—medial, intermediate, and lateral—providing a simple framework for classifying Lisfranc joint injuries. The medial column consists of the first metatarsal (M1) and the medial cuneiform (C1), the intermediate column includes the second and third metatarsals (M2–M3) together with the intermediate and lateral cuneiforms (C2–C3), and the lateral column comprises the fourth and fifth metatarsals (M4–M5) and the cuboid [2,4,7]. Furthermore, the joint is characterized by a mortise-like configuration in which the M2 fits tightly between the C1 and C3, acting as a keystone similar to Roman arches [6,7,8]. This structural feature contributes to stability during weight transfer by evenly transmitting body weight to the distal foot segments. The stability of the Lisfranc joint depends not only on the anatomical alignment of the bone structures but also on the integrity of the dorsal, interosseous, and plantar Lisfranc ligaments. Therefore, maintaining the morphological and functional integrity of the structures in this region is essential for preserving foot biomechanics [2,4,7,9].

Despite the inherent structural stability of the Lisfranc joint, injuries can still occur under various mechanical stresses. Lisfranc joint injuries are relatively uncommon, accounting for about 0.2% of all fractures; however, up to 24% may be missed on initial radiographs, indicating a higher true incidence [10,11]. They may occur as a result of falls, high-energy trauma such as vehicular accidents, or axial and rotational forces. The resulting damage is usually not confined to bone fractures but also involves the ligamentous structures. Cases involving only ligamentous structures with minimal radiological evidence are often challenging to diagnose clinically and tend to be overlooked. Consequently, untreated cases can lead to irreversible long-term conditions, including foot arch collapse, chronic pain, loss of function, and post-traumatic osteoarthritis [6,12,13].

Given the above considerations, it is essential to understand the detailed morphology of the Lisfranc joint for accurate diagnosis and effective treatment planning. However, most previous morphometric studies in the literature have been based either on cadaveric dissections or two-dimensional radiographic assessments, which may not fully capture the joint’s complex [2,14]. Although several studies have employed three-dimensional (3D) imaging techniques, these investigations have generally evaluated morphometric parameters in isolation, without integrating multiple structural features of the Lisfranc joint within a single comprehensive framework. Importantly, key aspects of joint morphology, including its mortise-like configuration and volumetric characteristics, have not been simultaneously assessed in a unified 3D analysis [15,16,17,18]. This highlights a significant gap in the literature, as addressing it could offer a more comprehensive anatomical perspective and improve the understanding of potential structural factors related to injury mechanisms. In this context, the present study aimed to provide a comprehensive morphometric evaluation of the bony anatomy of the Lisfranc joint using 3D computed tomography (CT) images, with particular emphasis on its mortise-like configuration, by integrating conventional measurements with an expanded set of parameters, and to investigate their relationship with Lisfranc joint injury. Previous studies have suggested that variations in joint morphology and structural configuration may influence joint stability and may be associated with Lisfranc joint injury [18,19]. Accordingly, we hypothesized whether specific morphometric characteristics of the Lisfranc joint are associated with injury occurrence.

## 2. Materials and Methods

### 2.1. Study Design and Ethical Approval

This study was designed as a retrospective case-control study using 3D CT images to evaluate the morphometric characteristics of the Lisfranc joint. The study was approved by the Acibadem Mehmet Ali Aydinlar University Medical Research Ethics Committee, with decision number 2023-13/45.

### 2.2. Study Population and CT Imaging

A total of 48 non-weight-bearing (NWB) CT images of the foot or ankle, obtained between 2009 and 2023 from individuals who presented to the Department of Orthopedics and Traumatology, were retrospectively included in the study. CT scans were acquired using a multidetector CT scanner (SOMATOM Force, Siemens Healthineers, Erlangen, Germany) with standard NWB lower-extremity CT protocols (120 kVp), a slice thickness of 0.5–1.0 mm, a 512 × 512 image matrix, and voxel sizes approximately 0.7 × 0.7 × 0.6 mm^3^.

The injury group consisted of 23 individuals, including 7 females and 16 males, with a mean age of 34.9 ± 12.3 years, who were radiologically diagnosed with Lisfranc joint injury. A distance of ≥ 2 mm between M1 and M2 is considered a radiographic sign of an unstable Lisfranc joint injury; hence, this parameter was used as the inclusion criterion for defining unstable Lisfranc joint injury in the injury group (Table 1). The exclusion criteria were severe dislocations or displaced fractures.

The control group consisted of 25 individuals, including 11 females and 14 males, with a mean age of 36.9 ± 12.4 years, whose CT scans were obtained for trauma or pain and showed no evidence of Lisfranc joint injury. The inclusion criteria were CT images without any fractures and/or dislocations of the Lisfranc joint complex. Cases with an M1–M2 distance of ≥ 2 mm without radiological evidence of Lisfranc joint injury were excluded from the control group to ensure a strictly defined healthy population (Table 1). CT images of individuals under 18 years of age were excluded from both groups. All CT images included in the study were anonymized.

### 2.3. Morphometric Analysis

#### 2.3.1. Image Processing and Standardization

Mimics^®^ (Version 21.0, Materialise NV, Leuven, Belgium, 2018) and ImageJ (Version 1.54g, National Institutes of Health, Bethesda, MD, USA, 2024) software were used for measurements. CT images in DICOM format were imported into Mimics software and converted into 3D images using the bone segmentation feature. For standardization, two reference planes were established: the transverse and sagittal. The transverse plane was defined to pass through the calcaneus and the heads of the first and fifth metatarsal bones. The sagittal plane was defined perpendicular to the transverse plane and passing through the navicular tuberosity.

#### 2.3.2. Mortise-like Configuration Parameters

**Figure 1:**
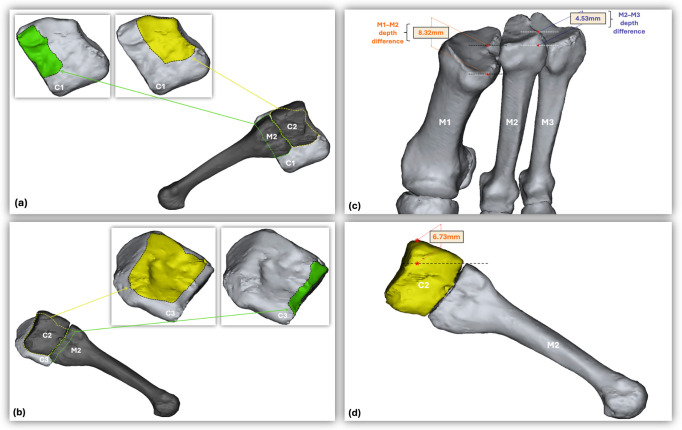
(**a**) Articular surfaces of the medial cuneiform (C1) with the second metatarsal (M2) and intermediate cuneiform (C2). (**b**) Articular surfaces of C1 with M2 and C2. (**c**) Demonstration of the depth difference measurement between the metatarsal bases (M1–M2 and M2–M3). (**d**) Demonstration of the dorsal step-off measurement, showing dorsal subluxation of M2 relative to C2.

To provide a detailed description of the mortise of the joint, the following measurements were performed: dorsal step off (dorsal subluxation of M2 relative to C2); depth differences between M1–M2 and M2–M3; articular surface area of C2 with M2; the articular surface area of C1 with M2; and the articular surface area of C3 with M2 (Figure 1).

#### 2.3.3. Additional Morphometric Parameters

**Figure 2:**
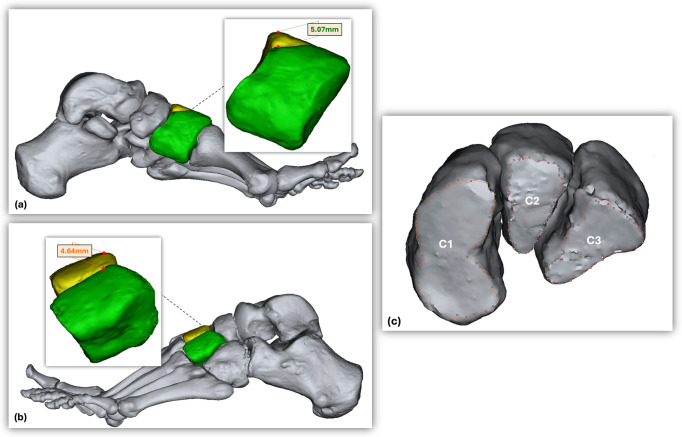
(**a**) Demonstration of the height difference measurements between the medial (C1) and intermediate (C2) cuneiforms (medial view; C1 in green, C2 in yellow). (**b**) Demonstration of the height difference measurements between the intermediate (C2) and lateral (C3) cuneiforms (lateral view; C3 in green, C2 in yellow). (**c**) Distal articular surfaces of the cuneiform bones.

In addition to the mortise-like configuration parameters, the following measurements were obtained: M2 length; the height difference between C1 and C2; the height difference between C2 and the C3; the articular surface area of C1 with M1; the articular surface area of C3 with M3; the articular surface area of C3 with C2; the articular surface area of C1 with C2; and the individual volumes of the cuneiform bones (Figure 2).

### 2.4. Statistical Analysis

Statistical analyses were performed using IBM SPSSTM (Version 24, IBM Corp., Armonk, NY, USA, 2016) and Microsoft Excel (Microsoft 365, Microsoft Corp., Redmond, WA, USA) software. All the parameters were compared between the control and injury groups. Correlations between M2 length and all parameters were evaluated separately in each group. Data conformity to normal distribution was assessed using the Shapiro-Wilk Test and box plot analysis. As all variables were normally distributed, parametric tests were applied throughout the analysis, and injury and control group measurements were compared using the independent-samples *t*-test. Relationships between variables were examined using Pearson correlation analysis. A 95% confidence interval was used in all analyses, and a *p*-value of <0.05 was considered statistically significant.

## 3. Results

The mean, standard deviation, minimum–maximum values, and two-tailed *p*-values for both the mortise-like configuration parameters and additional morphometric parameters in the control and injury groups are provided in Table 2 and Table 3. No statistically significant intergroup differences were observed in any of these parameters, suggesting that bony morphology alone may not be a distinguishing factor in Lisfranc joint injury.

A correlation analysis was performed between M2 length and morphometric parameters in the control group. Significant positive correlations were observed between M2 length and both the C1–C2 height difference and the M1–M2 depth difference. Regarding articular surface areas, strong positive correlations were identified between M2 length and the areas of the C1, C2, and C3. Similarly, the articular surface areas between C1–M2, C1–C2, and C3–C2 demonstrated significant positive associations with M2 length, whereas no significant correlation was found with the C3–M2 articular surface area. Regarding volumetric parameters, a high positive correlations were identified between M2 length and the volumes of C1, C2, and C3 (Table 4). These findings suggest that metatarsal length is proportionally related to joint surface area and bone volume within the Lisfranc complex, independent of injury status.

A correlation analysis was performed between M2 length and morphometric parameters in the injury group. Significant positive correlations were observed between M2 length and the articular surface areas of C1, C2, and C3. Furthermore, the articular surface areas between C1–C2 and C3–C2 were also significantly associated with M2 length. Regarding volumetric parameters, positive correlations were observed between M2 length and the volumes of C1, C2, and C3 (Table 5).

## 4. Discussion

This study offers a detailed 3D morphometric analysis of the Lisfranc joint, incorporating both conventionally reported measurements and an expanded set of parameters to characterize its anatomical structure. To the best of our knowledge, these parameters describing mortise morphology have not been previously reported in the literature. The findings demonstrated that these parameters can effectively describe the mortise-like configuration and provide a more comprehensive understanding of joint architecture. In addition, a significant positive correlation was identified between M2 length and several morphometric features, including articular surface areas and cuneiform volumes, suggesting a proportional relationship within the joint complex. However, no statistically significant differences were detected between the injury and control groups across any of the evaluated parameters. These findings suggest that bony architecture alone may not be a primary determinant of Lisfranc joint injury and highlight the potential importance of ligamentous and other non-osseous factors.

### 4.1. Evaluation of Mortise-like Configuration Parameters

The mortise-like configuration of the Lisfranc joint has been previously described as a key structural element that contributes to midfoot stability [6,7,8]. In the present study, this configuration was evaluated in detail using 3D reconstruction models, incorporating both conventionally reported measurements and newly defined parameters.

Among the parameters used to characterize this configuration, metatarsal depth differences have been frequently reported in the literature. De Luca et al. [20] and De Palma et al. [7] performed measurements of the M1–M2 depth difference on cadaver specimens in non-injured individuals. Similarly, Yu-Kai et al. [14] assessed the depth differences between M1–M2 and M2–M3 radiographically. In our study, which was also conducted in a healthy population, the measured depth differences were consistent with those reported in cadaveric data but were generally lower than values obtained from radiographic studies, suggesting a relatively shallower intermetatarsal configuration. These discrepancies may be attributed to methodological differences, including projection-related distortions in radiographic assessments, as well as variations in measurement techniques and specimen characteristics.

The articular surface area of C2 with M2 has been reported in the literature as an important parameter for characterizing the morphology of the Lisfranc joint, representing the central component of the Lisfranc articulation. Requist et al. [21] evaluated this area using micro-CT imaging of cadaveric specimens, whereas Clement et al. [22] performed the same measurement on CT images in non-injured populations. In the present study, also conducted in a healthy population, the measured surface areas were consistent with CT-based findings but slightly larger than those reported in cadaveric studies. This difference may be attributed to methodological factors, including tissue shrinkage and fixation-related changes in cadaveric specimens, as well as differences in imaging resolution and sample characteristics. This articulation represents the central load-bearing unit of the Lisfranc joint, and variations in its surface area may influence load distribution and joint congruency within the midfoot.

The dorsal step-off parameter, which assesses the dorsal subluxation of M2 relative to C2, is also among the key parameters used to describe the detailed mortise morphology of the Lisfranc joint. However, upon reviewing the literature, it was observed that there are inconsistencies among studies and a lack of standardized reference points for this measurement. For example, Bhimani et al. [23] performed their measurements on lateral CT images by measuring the distance between the highest point on the dorsal surface of M2 and the highest point on the distal surface of C2. In another study, de Bruijn et al. [18] measured the distance between the proximal corner of M2 and the distal corner of C2 on lateral radiographs. In our study, we evaluated this parameter by measuring the distance between the highest points on the dorsal surfaces of M2 and C2 in the medial view of the foot model, based on the reference planes we determined. Through this approach, this study contributes to the diversity of the literature by introducing an alternative measurement methodology based on 3D reconstruction modeling. The dorsal alignment between M2 and C2 plays an important role in maintaining joint congruency, and even small variations in this relationship may affect the distribution of forces across the Lisfranc joint.

Newly defined morphometric parameters, such as the articular surface area of C1 with M2 and the articular surface area of C3 with M2, were analyzed to better characterize the mortise configuration and to assess their potential contribution to joint stability. We propose that these specific parameters can reveal the morphological structure more thoroughly and may help clarify the role of structural factors in Lisfranc joint injuries. The interlocking configuration of M2 between C1 and C3 functions as a keystone within the Lisfranc joint, facilitating load transmission and contributing to overall joint stability. Accordingly, detailed evaluation of these parameters may provide further insight into the structural organization of the joint.

### 4.2. Evaluation of Additional Morphometric Parameters

Additional parameters reported in the literature for assessing the morphometry of the Lisfranc joint complex include the volumes of the cuneiform bones, as demonstrated by Çelik et al. [24], who evaluated each cuneiform individually in uninjured populations. Also, the articular surface areas of C1 with M1 and C3 with M3 were measured to provide a more detailed characterization of the Lisfranc joint morphology. Similar assessments have been reported in the literature: Requist et al. [21] evaluated the C1–M1 articular surface area in cadaveric samples using micro-CT imaging, and Clement et al. [22] conducted these analyses on clinical CT images. The volume measured for C1 in our study was slightly larger than the value reported by Çelik et al., while the other cuneiform volumes aligned with their findings. Our measurements of the articular surface area matched Requist et al.’s results but were lower than those reported by Clement et al.

In our study, in addition to the measurements frequently used in the literature, several novel parameters were obtained from CT images using a 3D modeling technique. These included the articular surface area of C1 with C2, the articular surface area of C3 with C2, and the height differences between C1–C2 and C2–C3. These parameters were incorporated to provide a more comprehensive evaluation of inter-cuneiform relationships and to better characterize the 3D organization of the Lisfranc joint.

### 4.3. Structural Correlates of Second Metatarsal Length in the Lisfranc Joint

Our analysis also included an additional parameter that has not been reported in previous studies: the potential correlation between M2 length and morphometric features of the Lisfranc joint. This analysis aimed to investigate whether variations in the metatarsal length are associated with corresponding changes in the morphological characteristics of the Lisfranc joint. No statistically significant correlation was found between M2 length and the dorsal step-off measurements in either group, nor with the depth or height differences of the cuneiform bones in the injury group. In contrast, a longer M2 was associated with a larger articular surface area between the cuneiforms and metatarsal bones in both groups. A positive correlation was also found between M2 length and the volumes of the cuneiform bones. These findings suggest a proportional relationship between metatarsal length and overall joint geometry, whereby a longer second metatarsal is accompanied by increased articular surface area and bone volume within the Lisfranc joint complex. This pattern may reflect coordinated morphological scaling within the midfoot rather than an isolated anatomical variation.

### 4.4. Comparison Between Control and Injury Groups

All morphometric parameters evaluated in this study were compared between the healthy and Lisfranc injury groups. Previous studies have also conducted comparative analyses; for example, Peicha et al. [19] compared the depth differences between metatarsals in both groups. They reported a statistically significant difference between groups in the M1–M2 depth measurement, indicating that the depth difference was smaller in the injury group, whereas no significant difference was observed for the M2–M3 measurement. In the present study, the depth differences between M1–M2 and M2–M3 showed no statistically significant difference between the two groups. Previous studies have suggested that M2 forms a shallower indentation in the injury group, which may increase the risk of Lisfranc joint injury. In contrast, our findings did not demonstrate a statistically significant association between indentation depth and Lisfranc joint injury. Furthermore, while previous investigations have shown significantly increased dorsal step-off values in the injury group compared to healthy [18,23], our findings indicate a similar dorsal alignment between C2 and M2 in the control and injury groups. This comprehensive 3D morphometric assessment of the Lisfranc joint indicates that intrinsic bony anatomy alone is unlikely to represent a primary predisposing factor for Lisfranc injuries.

The main factor contributing to the discrepancies between our findings and previous reports is thought to be the variation in imaging modalities employed. CT has been shown to be more sensitive and diagnostically superior to conventional radiography in detecting subtle subluxations, non-displaced or occult fractures, and minor misalignments within the Lisfranc joint [6,25]. Accordingly, CT imaging was preferred as the primary modality in our study, and the images were reconstructed in three dimensions. The use of 3D reconstruction techniques for the morphometric evaluation of the Lisfranc joint remains limited in the current literature. Bhimani et al. [23] employed this method only for volumetric analysis, while other parameters were assessed in two dimensions. In contrast, in our study, length and articular surface area measurements were performed for the first time on 3D foot models reconstructed from CT data, allowing for a more detailed and accurate assessment of bone morphology. Furthermore, while most studies in the existing literature have focused on cases of significant displacement of fractures or dislocations, our study included patients with partial instability due to ligamentous injury, excluding those with severe dislocation or displaced fractures. This selection criterion may have contributed to the absence of significant differences in bone morphometry between the injury and control groups. These findings highlight the influence of the clinical characteristics of the patient group on the morphometric outcomes obtained. In this regard, our research, which includes both patient and control groups and employs more precise measurement techniques such as 3D modeling, has the potential to provide more detailed and comparative data than traditional methods reported in the literature. Accordingly, our study offers a methodological contribution to the existing literature and may serve as a foundation for future comprehensive research; however, the present findings indicate that bony morphological parameters alone may not be sufficient to distinguish Lisfranc joint injuries. Therefore, greater emphasis should be placed on ligamentous integrity and functional assessment in clinical evaluation.

### 4.5. Limitations

The relatively small sample size may have limited the statistical power to detect subtle differences between groups. This limitation is partly due to the clinical difficulty in diagnosing Lisfranc joint injuries and the challenges associated with assembling a larger cohort. In addition, all measurements were performed using NWB CT images without comparison to weight-bearing imaging. Given that weight-bearing imaging better reflects functional joint alignment and has been shown to improve the detection of subtle Lisfranc instability [18,26,27], the use of NWB imaging may have contributed to the lack of significant differences observed between groups. Another important limitation is that the present study focused exclusively on bony morphology and did not include an assessment of ligamentous structures. As Lisfranc injuries may occur in the absence of significant bony abnormalities due to ligament involvement [28], the lack of ligament evaluation may have limited the ability to fully characterize joint stability and structural integrity within the Lisfranc complex.

## 5. Conclusions

This study assessed the morphometric characteristics of the Lisfranc joint complex using a 3D reconstruction technique on CT imaging, providing detailed insights into the joint’s bony morphology. No significant association was identified between bony morphology and Lisfranc joint injury. Metatarsal length showed a positive correlation with morphometric parameters, including joint surface area and bone volume, independent of injury status. These results indicate that bony morphology alone may not be a primary determinant of Lisfranc joint injury, suggesting that assessment based solely on osseous structures may be insufficient, and highlighting the potential importance of ligamentous and other non-osseous structures in the underlying injury mechanisms. Future studies incorporating both osseous and soft tissue components may provide a more comprehensive understanding of Lisfranc joint pathology.

## Figures and Tables

**Table 1:** M1–M2 distance between groups.

	Control Group		Injury Group	
**Parameter**	Mean ± SD	Min–Max	Mean ± SD	Min–Max
M1–M2 distance (mm)	1.34 ± 0.45	0.33–1.99	2.78 ± 0.58	2.12–4.37

All data are given as mean ± SD (min–max). SD: standard deviation.

**Table 2:** Mortise-Like Configuration Parameters: Comparison Between Control and Injury Groups.

**Parameters**	Control Group	Injury Group	*p* Value
	Mean ± SD (Min–Max)	Mean ± SD (Min–Max)	
Dorsal step-off (mm)	6.88 ± 1.08 (4.27–8.88)	6.73 ± 1.71 (2.99–9.46)	0.72
M1–M2 depth difference (mm)	8.49 ± 1.93 (4.84–11.82)	8.22 ± 2.34 (4.59–12.87)	0.67
M2–M3 depth difference (mm)	4.02 ± 1.54 (0.62–6.09)	4.33 ± 1.10 (2.21–6.69)	0.42
C2–M2 articular surface area (mm^2^)	167.74 ± 32.79 (104.33–244.19)	172.01 ± 30.46 (111.50–223.10)	0.64
C1–M2 articular surface area (mm^2^)	184.27 ± 37.84 (107.27–265.95)	184.38 ± 39.91 (87.39–257.77)	0.99
C3–M2 articular surface area (mm^2^)	46.38 ± 19.66 (3.19–87.23)	55.45 ± 57.59 (7.30–89.13)	0.46

All data are given as mean ± SD (min–max). SD: standard deviation.

**Table 3:** Additional Morphometric Parameters: Comparison Between Control and Injury Groups.

**Parameters**	Control Group	Injury Group	*p* Value
	Mean ± SD (Min–Max)	Mean ± SD (Min–Max)	
M2 length (mm)	63.00 ± 6.21 (55.10–75.50)	61.84 ± 4.55 (50.13–70.48)	0.46
C1–C2 height difference (mm)	5.55 ± 1.44 (2.99–7.88)	5.36 ± 1.62 (2.83–9.15)	0.67
C2–C3 height difference (mm)	4.59 ± 1.35 (1.16–6.95)	5.47 ± 1.90 (1.22–8.92)	0.07
C1–M1 articular surface area (mm^2^)	351.54 ± 67.82 (264.92–514.45)	345.53 ± 63.30 (220.96–476.66)	0.75
C3–M3 articular surface area (mm^2^)	182.52 ± 37.77 (104.84–262.26)	190.11 ± 35.70 (119.21–248.89)	0.47
C1–C2 articular surface area (mm^2^)	318.91 ± 59.13 (218.60–483.16)	326.55 ± 54.41 (236.23–437.34)	0.64
C3–C2 articular surface area (mm^2^)	265.64 ± 63.33 (163.73–432.83)	250.52 ± 50.73 (136.86–339.24)	0.36
C1 volume (mm^3^)	10,144.06 ± 2797.00 (6471.33–17,855.09)	10,416.73 ± 1721.93 (7.904.07–14.319.63)	0.68
C2 volume (mm^3^)	3917.65 ± 1059.53 (2199.12–6808.33)	4092.33 ± 778.85 (2786.45–5733.68)	0.52
C3 volume (mm^3^)	5664.89 ± 1603.00 (3035.03–9612.57)	5720.49 ± 1116.57 (4156.31–7642.26)	0.89

All data are given as mean ± SD (min–max). SD: standard deviation.

**Table 4:** Correlations Between M2 Length and Morphometric Parameters in the Control Group.

Parameters	M2 Length	
	Pearson Correlation Coefficient (r)	*p* Value
M1–M2 distance (mm)	−0.02	0.896
C1–C2 height difference (mm)	0.44	**0.025**
C2–C3 height difference (mm)	0.14	0.483
Dorsal step-off (mm)	0.04	0.820
M1–M2 depth difference (mm)	0.56	**0.004**
M2–M3 depth difference (mm)	0.29	0.154
C1–M1 articular surface area (mm^2^)	0.70	**<0.001**
C2–M2 articular surface (mm^2^)	0.75	**<0.001**
C3–M3 articular surface (mm^2^)	0.65	**<0.001**
C1–M2 articular surface (mm^2^)	0.61	**0.001**
C1–C2 articular surface (mm^2^)	0.75	**<0.001**
C3–M2 articular surface (mm^2^)	0.25	0.218
C3–C2 articular surface (mm^2^)	0.59	**0.002**
C1 volume (mm^3^)	0.75	**<0.001**
C2 volume (mm^3^)	0.74	**<0.001**
C3 volume (mm^3^)	0.76	**<0.001**

Bold values indicate statistically significant results (*p* < 0.05).

**Table 5:** Correlations Between M2 Length and Morphometric Parameters in the Injury Group.

Parameters	M2 Length	
	Pearson Correlation Coefficient (r)	*p* Value
M1–M2 distance (mm)	−0.25	0.244
C1–C2 height difference (mm)	0.37	0.076
C2–C3 height difference (mm)	−0.12	0.562
Dorsal step-off (mm)	−0.00	0.974
M1–M2 depth difference (mm)	0.19	0.385
M2–M3 depth difference (mm)	0.01	0.951
C1–M1 articular surface area (mm^2^)	0.58	**0.004**
C2–M2 articular surface (mm^2^)	0.47	**0.024**
C3–M3 articular surface (mm^2^)	0.58	**0.004**
C1–M2 articular surface (mm^2^)	0.35	0.096
C1–C2 articular surface (mm^2^)	0.56	**0.005**
C3–M2 articular surface (mm^2^)	0.29	0.173
C3–C2 articular surface (mm^2^)	0.52	**0.011**
C1 volume (mm^3^)	0.64	**0.001**
C2 volume (mm^3^)	0.55	**0.006**
C3 volume (mm^3^)	0.61	**0.002**

Bold values indicate statistically significant results (*p* < 0.05).

## Data Availability

The original contributions presented in this study are included in the article. Further inquiries can be directed to the corresponding author.

## Abbreviations

The following abbreviations are used in this manuscript:

TMT	Tarsometatarsal
M1	First metatarsal
M2	Second metatarsal
C1	Medial cuneiform
C2	Intermediate cuneiform
C3	Lateral cuneiform
3D	Three-dimensional
CT	Computed tomography
NWB	Non-weight-bearing

## References

[1] Chaney D.M. (2010). The Lisfranc joint. Clin. Podiatr. Med. Surg..

[2] Castro M., Melão L., Canella C., Weber M., Negrão P., Trudell D., Resnick D. (2010). Lisfranc joint ligamentous complex: MRI with anatomic correlation in cadavers. AJR Am. J. Roentgenol..

[3] Burroughs K.E., Reimer C.D., Fields K.B. (1998). Lisfranc injury of the foot: A commonly missed diagnosis. Am. Fam. Physician.

[4] Johnson A., Hill K., Ward J., Ficke J. (2008). Anatomy of the Lisfranc ligament. Foot Ankle Spec..

[5] Hardcastle P., Reschauer R., Kutscha-Lissberg E., Schoffmann W. (1982). Injuries to the tarsometatarsal joint. Incidence, classification and treatment. J. Bone Jt. Surg. Br. Vol..

[6] Moracia-Ochagavía I., Rodríguez-Merchán E.C. (2019). Lisfranc fracture-dislocations: Current management. EFORT Open Rev..

[7] de Palma L., Santucci A., Sabetta S.P., Rapali S. (1997). Anatomy of the Lisfranc joint complex. Foot Ankle Int..

[8] Llopis E., Carrascoso J., Iriarte I., Serrano M.d.P., Cerezal L. (2016). Lisfranc Injury Imaging and Surgical Management. Semin. Musculoskelet. Radiol..

[9] Mason L., Jayatilaka M.L.T., Fisher A., Fisher L., Swanton E., Molloy A. (2020). Anatomy of the Lateral Plantar Ligaments of the Transverse Metatarsal Arch. Foot Ankle Int..

[10] Rossi M., Wallace G.F. (2021). The piano key test: When and How, a survey. Foot Ankle Surg. Tech. Rep. Cases.

[11] Seow D., Yasui Y., Chan L.Y.T., Murray G., Kubo M., Nei M., Matsui K., Kawano H., Miyamoto W. (2023). Inconsistent radiographic diagnostic criteria for lisfranc injuries: A systematic review. BMC Musculoskelet. Disord..

[12] Chen J., Sagoo N., Panchbhavi V.K. (2021). The Lisfranc Injury: A Literature Review of Anatomy, Etiology, Evaluation, and Management. Foot Ankle Spec..

[13] Cenatiempo M., Buzzi R., Bianco S., Iapalucci G., Campanacci D.A. (2019). Tarsometatarsal joint complex injuries: A study of injury pattern in complete homolateral lesions. Injury.

[14] Yu-Kai Y., Shiu-Bii L. (2015). Anatomic Parameters of the Lisfranc Joint Complex in a Radiographic and Cadaveric Comparison. J. Foot Ankle Surg..

[15] Myerson M.S. (1999). The diagnosis and treatment of injury to the tarsometatarsal joint complex. J. Bone Jt. Surg. Br..

[16] Watson T.S., Shurnas P.S., Denker J. (2010). Treatment of Lisfranc joint injury: Current concepts. J. Am. Acad. Orthop. Surg..

[17] Grewal U.S., Onubogu K., Southgate C., Dhinsa B.S. (2020). Lisfranc injury: A review and simplified treatment algorithm. Foot.

[18] De Bruijn J., Hagemeijer N.C., Rikken Q.G.H., Husseini J.S., Saengsin J., Kerkhoffs G., Waryasz G., Guss D., DiGiovanni C.W. (2022). Lisfranc injury: Refined diagnostic methodology using weightbearing and non-weightbearing radiographs. Injury.

[19] Peicha G., Labovitz J., Seibert F.J., Grechenig W., Weiglein A., Preidler K.W., Quehenberger F. (2002). The anatomy of the joint as a risk factor for Lisfranc dislocation and fracture-dislocation. An anatomical and radiological case control study. J. Bone Jt. Surg. Br..

[20] DeLuca M.K., Boucher L.C. (2023). Morphology of the Lisfranc joint complex. J. Foot Ankle Surg..

[21] Requist M.R., Rolvien T., Barg A., Lenz A.L. (2023). Morphologic analysis of the 1st and 2nd tarsometatarsal joint articular surfaces. Sci. Rep..

[22] Clements J.R., Whitmer K., Nguyen H., Rich M. (2018). Cross-Sectional Area Measurement of the Central Tarsometatarsal Articulation: A Review of Computed Tomography Scans. J. Foot Ankle Surg..

[23] Bhimani R., Sornsakrin P., Ashkani-Esfahani S., Lubberts B., Guss D., De Cesar Netto C., Waryasz G.R., Kerkhoffs G., DiGiovanni C.W. (2021). Using area and volume measurement via weightbearing CT to detect Lisfranc instability. J. Orthop. Res..

[24] Gurlek Celik N., Akman B. (2024). Morphological and morphometric analysis of tarsal bones according to sex. Surg. Radiol. Anat..

[25] Sripanich Y., Weinberg M.W., Krähenbühl N., Rungprai C., Mills M.K., Saltzman C.L., Barg A. (2020). Imaging in Lisfranc injury: A systematic literature review. Skelet. Radiol..

[26] Sripanich Y., Weinberg M.W., Krähenbühl N., Rungprai C., Saltzman C.L., Barg A. (2021). Reliability of measurements assessing the Lisfranc joint using weightbearing computed tomography imaging. Arch. Orthop. Trauma Surg..

[27] Talaski G.M., Baumann A.N., Walley K.C., Anastasio A.T., de Cesar Netto C. (2023). Weightbearing Computed Tomography vs Conventional Tomography for Examination of Varying Degrees of Lisfranc Injures: A Systematic Review of the Literature. Foot Ankle Orthop..

[28] Nunley J.A., Vertullo C.J. (2002). Classification, investigation, and management of midfoot sprains: Lisfranc injuries in the athlete. Am. J. Sports Med..

